# Outcome and Post-Surgical Lung Biopsy Change in Management of ARDS: A Proportional Prevalence Meta-Analysis

**DOI:** 10.3390/arm90040036

**Published:** 2022-07-28

**Authors:** Tanveer Mir, Neelambuj Regmi, Ghulam Saydain, Viren Kaul, Ayman O. Soubani, Waqas T. Qureshi

**Affiliations:** 1 Internal Medicine, Wayne State University, Detroit Medical Center, Detroit, MI 48201, USA; gr6723@wayne.edu; 2 Pulmonary Critical Care, Wayne State University, Detroit Medical Center, Detroit, MI 48201, USA; nregmi@med.wayne.edu (N.R.); gsaydain@med.wayne.edu (G.S.); 3 Pulmonary Critical Care, Crouse Health, Syracuse, NY 13210, USA; jishuviren@gmail.com; 4 School of Medicine, University of Massachusetts, Worcester, MA 01655, USA; waqas.qureshi@umassmed.edu

**Keywords:** ARDS, open lung biopsy, change in management, mortality

## Abstract

**Highlights:**

- Lung biopsy in ARDS patients lead to a change in management in 3/4 patients.
- Despite changes in management and pathology-directed treatment, there was significant mortality in ARDS patients who had lung biopsy.
- High rates of complications from lung biopsy were observed in ARDS patients who had lung biopsy.

**Abstract:**

Background: Limited epidemiological data are available on changes in management, benefits, complications, and outcomes after open lung biopsy in patients with ARDS. Methods: We performed a literature search of PubMed, Ovid, and Cochrane databases for articles from the inception of each database till November 2020 that provided outcomes of lung biopsy in ARDS patients. The primary outcome was the proportion of patients that had a change in management with alteration of treatment plan, after lung biopsy. Secondary outcomes included pathological diagnoses and complications related to the lung biopsy. Pooled proportions with a 95% confidence interval (CI) were calculated for the prevalence of outcomes. Results: After analysis of 22 articles from 1994 to 2018, a total of 851 ARDS patients (mean age 59.28 ± 7.41, males 56.4%) that were admitted to the ICU who underwent surgical lung biopsy for ARDS were included. Biopsy changed the management in 539 patients (pooled proportion 75%: 95% CI 64–84%). There were 394 deaths (pooled proportion 49%: 95% CI 41–58%). The most common pathologic diagnosis was diffuse alveolar damage that occurred in 30% (95% CI 19–41%), followed by interstitial lung disease in 10% (95% CI 3–19%), and viral infection in 9% (95% CI 4–16%). Complications occurred among 201 patients (pooled proportion 24%, 95% CI 17–31%). The most common type of complication was persistent air-leak among 115 patients (pooled estimate 13%, 95% CI 9–17%). Conclusion: Despite the high mortality risk associated with ARDS, lung biopsy changed management in about 3/4 of the patients. However, 1/4 of the patients had a complication due to lung biopsy. The risks from the procedure should be carefully weighed before proceeding with lung biopsy.

## 1. Introduction

Acute respiratory distress syndrome (ARDS) is a life-threatening form of respiratory failure characterized by widespread inflammation of the lung parenchyma [1]. ARDS constitutes an estimated 10.4% of the intensive care unit (ICU) patients and 23.4% of the ventilated patients with an estimated in-hospital mortality of 34–55% [2,3]. The Berlin definition for ARDS includes bilateral opacities on a chest radiograph with the partial pressure of oxygen to fraction of inspired oxygen ratio ≤300 mm Hg [4]. Diffuse alveolar damage (DAD) is often considered as the histological hallmark of ARDS. Autopsy results of lung biopsies by Thille et al. showed that only 45% of the 356 patients had DAD that met the Berlin definition of ARDS [5]. Organizing pneumonia as an adverse reaction to drugs and hypersensitivity pneumonitis are other etiologies of ARDS [6].

In most cases, a definite etiology can be ascertained by imaging, microbiologic studies, and thorough history and physical examination. However, due to similar-appearing clinical and radiological presentation, the etiologic diagnosis can be difficult in some cases of ARDS. For such patients where ARDS persists and the etiology is unclear despite diagnostic evaluation by minimally invasive techniques including bronchoscopy, open lung biopsy (OLB) is a consideration. A study by Laura et al. suggested lung biopsy as an option for such patients; however, the patient population in the study was not limited to patients fulfilling the Berlin criteria [7]. There also have been multiple previous studies showing the benefits of OLB in patients with ARDS. A study by Papazion et al. in 2007 showed that the patient with a contributive diagnosis and that underwent OLB had improved survival [8]. Additionally, there have been studies showing the benefit of steroids, especially in the fibroproliferative phase [9,10]. Hence, the role of biopsy in patients with ARDS is to identify the fibrosis as well as rule out any other potential infections, especially fungal infections that are not typically covered by empiric antibiotics. We aimed to conduct a proportion meta-analysis for studies of open lung biopsy in ARDS patients to evaluate the utility and safety of open lung biopsy in the intensive care unit (ICU) setting.

## 2. Methods

### 2.1. Search Strategy

**Figure 1:**
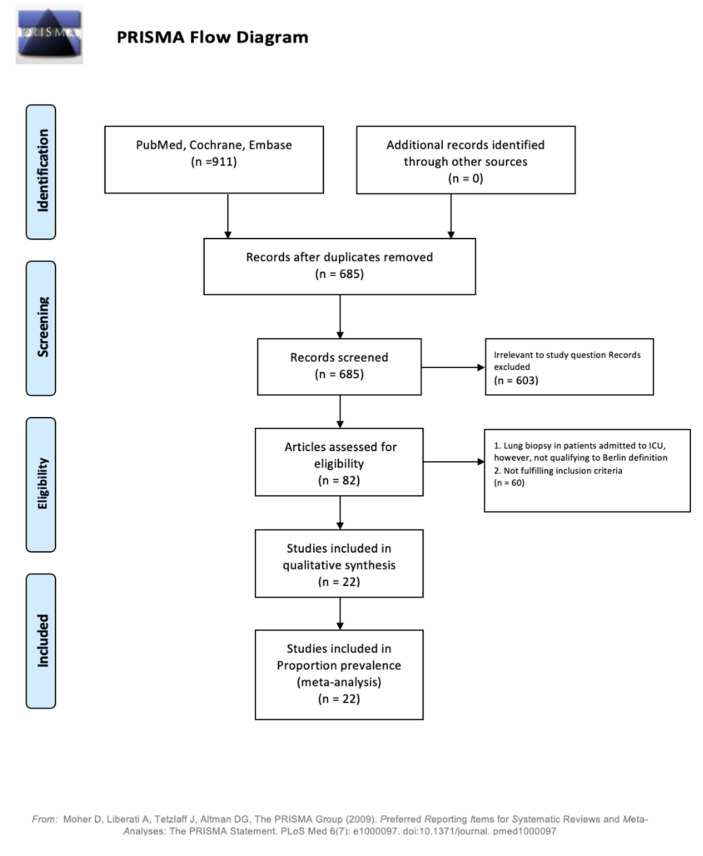
Demographics and baseline characteristics of the studies. n: number; SD: standard deviation. PEEP: peak end-expiratory pressure; PaO_2_/FiO_2_: partial pressure of arterial oxygen/fraction (percent) of inspired oxygen. Flow diagram based on [11].

The meta-analysis was performed using the standard protocol devised by the “Meta-analysis of Observational Studies in Epidemiology (MOOSE)”. Electronic databases including PubMed, Ovid, Cochrane, and Clinicaltrial.org were searched using a combination of medical subject headings and key terms such as “ARDS”, “open lung biopsy”, “outcome”, and “change in management”. A cross-reference check of previously published articles on this topic was also performed. The full text of potentially relevant articles was read by the two authors (T.M. and N.R.). Disagreements were resolved by consensus. All extracted data from the included studies was verified by a third author (V.K.). The search was restricted to the English literature published from the inception of a database till November 2020. Only studies that included patients more than 18 years of age who were admitted to the critical care unit for ARDS as per the Berlin definition and evaluated for the pathological outcome of lung biopsy, stated a change in management after lung biopsy, and detailed complications secondary to lung biopsy were included [4]. Studies with insufficient data, case reports, duplicate data, and review articles were excluded. We excluded studies evaluating open lung biopsy in patients not fulfilling the Berlin criteria for ARDS [4]. The Preferred Reporting Items for Systematic Reviews and Meta-Analyses (PRISMA) were followed to obtain studies for quantitative analysis (Figure 1).

### 2.2. Data Extraction

Data were collected for: (1) baseline characteristics of age, sex, baseline oxygenation requirements; (2) pathological outcomes of lung biopsy; (3) change in management after lung biopsy and complications from biopsy; (4) all-cause mortality. The baseline characteristics are given in Table 1. All outcomes were studied during the hospital stay. The method of open lung biopsy was mostly open thoracotomy and 2 studies reported video-assisted thoracotomy in 13 out of 72 patients [12,13].

### 2.3. Outcomes

The primary outcome was the proportion change in management in terms of change in treatment based on the lung biopsy results and mortality. Secondary outcomes included pathological diagnosis and complications from lung biopsy.

### 2.4. Statistical Analysis

Baseline characteristics were obtained from a review of the included studies. Continuous variables from the studies were pooled and mean estimates along with standard errors were calculated using Metan in Stata 16.0. Pooled estimates of the prevalence rates for pathology results from the lung biopsy of patients with ARDS, change in management, complications from lung biopsy, and mortality outcomes were observed in the 22 studies. All analyses were carried out using Stata (version 16.0, StataCorp LLC, College Station, TX, USA). Meta-analysis was performed adopting a specific Stata module, Metaprop, designed to perform meta-analyses of proportions in Stata [33]. A random effects model was used for calculation of the prevalence. The random effects model assumes that the studies included in the meta-analysis are a random sample of hypothetical study populations. The estimated effect size was reported as a point estimate and 95% confidence interval (CI). An alpha criterion of a *p*-value less than 0.05 was considered statistically significant. The Higgins I-squared (I^2^) statistical model was used to evaluate variations in outcomes of included studies. I^2^ values of 50% or less corresponded to low to moderate, and 75% or higher indicated large amounts of heterogeneity. A meta-regression test using Stata 16 was conducted to rule out the confounding effect of common variables such as age, gender, and study types for the outcome. Results are reported as regression coefficients, alfa and beta coefficients of the regression equation with 95% CIs, and two-sided *p*-values. The methodological quality was performed by screening all included articles for different types of biases (selection, representation, exposure and outcome adequacy, causality, and reporting) and evaluated as per the modified tool for a quality assessment of the case series [34].

## 3. Results

### 3.1. Search Results and Study Characteristics

An initial search on multiple databases identified 911 articles. After exclusion of duplicates (226) and the articles irrelevant to the study question (631), 82 studies were deemed relevant for full-text review. Next, 60 articles were excluded as a lung biopsy was performed on patients not fulfilling the Berlin criteria for ARDS and not within the inclusion criteria. Ultimately, 22 studies qualified for quantitative analysis: 20 studies were retrospective studies [14,15,16,17,18,19,20,21,22,23,24,26,27,28,29,30,31,32] and 2 studies were prospective studies [8,25] The detailed PRISMA flow diagram is shown in Figure 1.

### 3.2. Primary Outcomes

**Figure 2:**
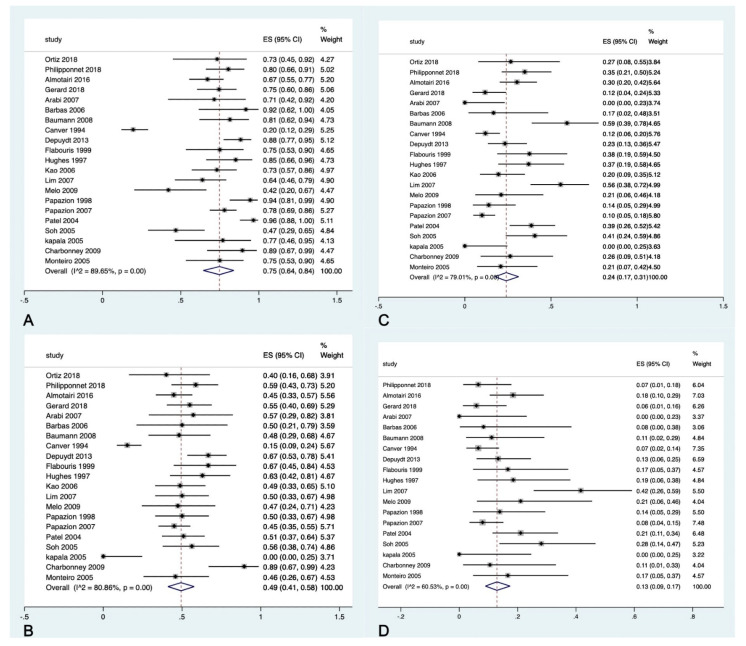
(**A**) Pooled proportion for change in management after lung biopsy. (**B**) Pooled prevalence for mortality results for ARDS patients who had open lung biopsy. (**C**) Pooled prevalence of complication rate after open lung biopsy. (**D**) Prevalence proportion for air-leak [14,15,16,17,18,19,20,21,22,23,24,25,26,27].

A total of 851 ARDS patients (mean age 59.28 ± 7.41, males 56.4%) that underwent biopsy were included. There was a change in management after lung biopsy in 539 patients (pooled proportion 75%: 95% CI 64–84%, Figure 2A). The most common change in management was a change in antibiotic treatment, which was observed in 2/3 of the total patients. Despite the change in management, half of the admitted patients died during the hospital course, which amounted to 394 deaths (pooled proportion 49%: 95% CI 41–58%, Figure 2B).

Table 2 Results of regression analysis. Univariate and multivariate regression to evaluate confounding effects of age, gender, and study type. CIM: change in management.

### 3.3. Secondary Outcomes

#### 3.3.1. Complications

Complications occurred among 201 patients (pooled proportion 24%, 95% CI 17–31%, Figure 2C). The most common type of complication was persistent air-leak among 115 patients (pooled estimate 13%, 95%CI 9–17%, Figure 2D).

##### Regression Analysis

**Figure 3:**
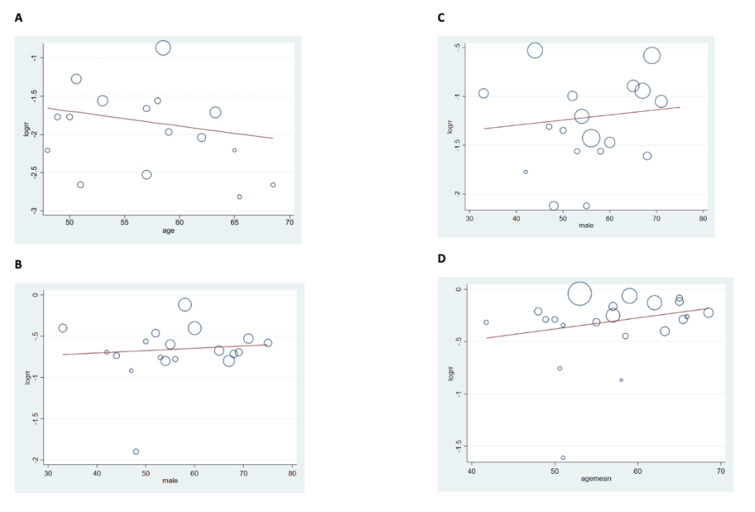
Univariate regression to evaluate confounding effects of age, gender, and study type on results. (**A**) Regression plot evaluating the effects of age on persistent air-leak; (**B**) regression plot evaluating the effects of gender on mortality; (**C**) regression plot evaluating the effects of gender on complication rate; (**D**) regression plot evaluating the effects of age on change in management.

To examine the influence of age, gender, and study type on results, we did a regression analysis. No confounding effects were seen secondary to the variable in the results from the univariate and multivariate regression analyses. Table 2. (Figure 3, Supplementary File).

#### 3.3.2. Pathologic Diagnosis

**Figure 4:**
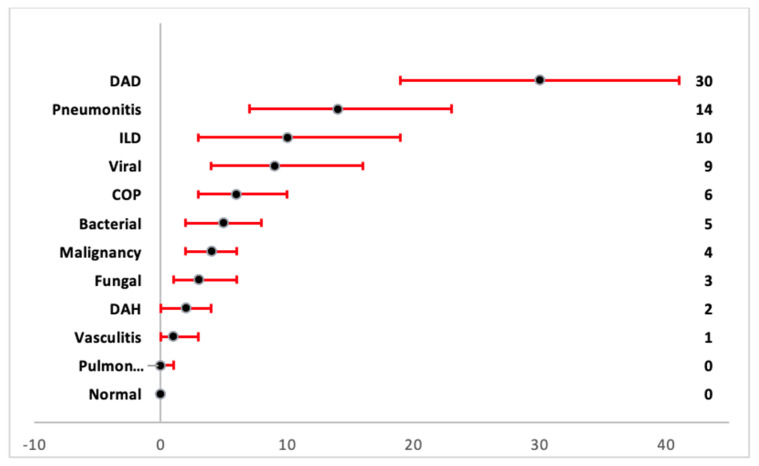
Forest plot for pooled proportion with 95% CI of pathology results. DAD was most common pathology reported in lung biopsy results. DAD: diffuse alveolar damage; COPD: chronic obstructive pulmonary disease; DAH: diffuse alveolar hemorrhage; ILD: interstitial lung disease. pulmon = pulmonary edema.

**Figure 5:**
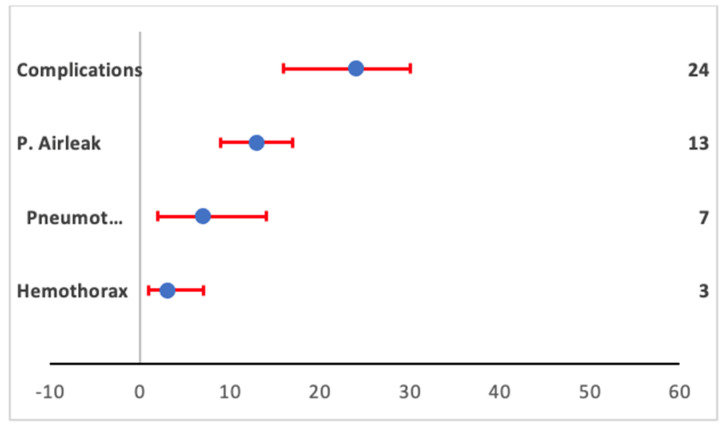
Forest plot for pooled proportion with 95% CI of complication rate and various complications. Persistent air-leak was most common, followed by pneumothorax, and least common was hemothorax. P. airleak: persistent air-leak, pneumot: pneumothorax.

**Figure 6:**
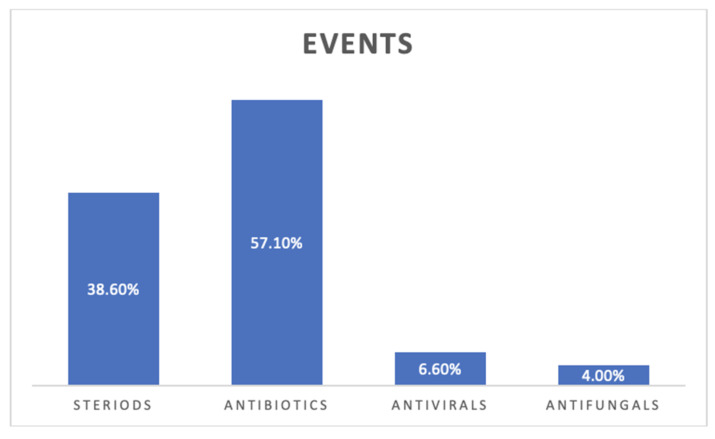
Change in treatment percentage after lung biopsy represented in the bar diagram. Most common changes were seen in antibiotic management (antibiotics were either stopped or changed to different antibiotics), followed by change in steroid management and antivirals, and least common were antifungal management changes.

Diffuse alveolar damage was the predominant pathological diagnosis from the biopsy results, with a pooled proportion of 30% (95% CI 19–41%). Among patients with etiologic diagnosis from lung biopsies, viral etiology was observed in 9% (95% CI 4–16%), bacterial in 5% (95% CI 2–8%), and fungal in 3% (95% CI 1–6%). Among non-infectious etiologies, cryptogenic organizing pneumonia had a prevalence of 6% (95% CI 3–10%), interstitial lung disease 10% (95% CI 3–19%), vasculitis 1% (95% CI 0–3%), pneumonitis 14% (95% CI 7–23%), diffuse alveolar hemorrhage 2% (95% CI 0–4%), and malignancy 4% (95% CI 2–6%). Pulmonary edema and normal pathology results had a proportion prevalence of 0% (95% CI 0–1%) and 0% (95% CI 0–0%), respectively. Forest plots of pathology results are presented in the Supplementary File. The percentage of prevalence for the pathological outcome is demonstrated in the forest plot in Figure 4. Among the complications, other than persistent air-leak, hemothorax had a prevalence of 3% (95% CI 1–7%) and pneumothorax had a prevalence of 7% (95% CI 2–14%) (Supplementary File). The complications secondary to lung biopsy are given in the forest plot in Figure 5. The change in management in terms of change in treatment is displayed as a bar diagram in Figure 6.

##### Quality of the Included Studies

We included 20 retrospective [27,29] and 2 prospective case studies [8,25]. The quality assessment was performed using the modified tool for quality assessment of case series [34]. The studies were assessed for selection, representation, exposure, and outcome adequacy, causality, and reporting. The mean score of the included studies was 6. The overall quality of included studies was moderate (Supplementary File).

## 4. Discussion

In this case of a series-based meta-analysis of ARDS patients in the ICU, the pathological results of lung biopsy led to changes in management in approximately 3/4 of the patients. The most common pathological diagnosis was diffuse alveolar damage. Among the infectious etiologies, viral infections were the most common cause of ARDS, followed by bacterial and fungal infections. The most common complication was persistent air-leak after lung biopsy, noted in up to 1/7 of the patients. Despite confirmation of diagnosis and change in management, half of the patients died.

### 4.1. High Risk of Mortality with Lung Biopsy in ARDS

Prior studies reported in-hospital and ICU mortality rates for ARDS of 35.3% and 40%, respectively [3]. Studies have reported higher mortality in patients with an exacerbation of interstitial lung disease. Miyazaki et al. reported a mortality rate of 85.7% in patients who had experienced an acute exacerbation of interstitial lung disease [35]. A meta-analysis of 11 studies involving 949 patients reported mortality of 44% in patients with ARDS [36]. However, our analysis notes a much higher mortality (~49%). This could be a result of the use of an invasive surgical procedure, the lung biopsy, in these already critically ill patients. Alternatively, this could be also related to the fact that these patient represented a sicker cohort that was not making clinical progress, necessitating the use of an interventional procedure to tease out the etiology. It was reported that mortality rates are higher in patients with a non-elective lung biopsy (16%) than elective biopsy (1.7%) [37]. The high prevalence of ILD (10%) could be another reason for high mortality in our study.

### 4.2. Pathological Diagnoses on Lung Biopsy and Change in Management

In up to 30% of the patients, pathological analysis of the lung biopsy did not lead to a specific etiological diagnosis, but rather demonstrated a histological diagnosis of diffuse alveolar damage, known to be the hallmark of ARDS. Even though the most common cause of diffuse alveolar damage is pneumonia, [38] infectious etiologies were only identified in 1/6 of the patients with a viral diagnosis as the most common diagnosis found on biopsy. This is in line with the meta-analysis of 512 patients reported by Wong et al., that reported a 20% prevalence of infections, with viral infections being the most common causes, followed by bacterial and fungal infections [39]. The most common non-infectious diagnosis was pneumonitis, followed by interstitial lung disease and malignancy. Our study reported a low prevalence proportion for DAH (2%) and vasculitis (1%). The lung biopsy results led mostly to a change in antibiotic regimen or the introduction of steroids. After lung biopsy, the management change in terms of antibiotic treatment was observed in 2/3 of total patients. The change in antibiotic management was either a discontinuation of the antibiotics or a change to a different class of antibiotics. This might be secondary to high rates of empiric antibiotics given to ARDS patients. However, these changes did not translate into better mortality in this case series.

### 4.3. High Complication Rate with Lung Biopsy

We noted complications in up to 1/4 of the patients. This is likely due to under-utilization of video-assisted thoracoscopic surgery. Bensard et al., in their study of 21 thoracotomies vs. 22 video-assisted thoracoscopic biopsies, reported higher complications with thoracotomy procedures (19% vs. 9%, respectively), with the most common being an air-leak from bronchopleural fistula [40]. Our study revealed a high proportion prevalence of 13%. for persistent air-leak. We postulate that using video-assisted thoracoscopy and biopsy could improve the complication rates in this population of patients as compared to thoracotomy. However, there are currently no guidelines regarding this, and there is a need for further research in this area.

There are several implications of this study. First, this study provides us with the landscape of the current practice of lung biopsy in patients with ARDS. Most patients with an unknown etiology of ARDS undergo open lung biopsy. Second, we observed a significant mortality rate among these patients, which could be due to the severity of the illness or related to the invasive surgical procedure. Third, the complication rate was high amongst these patients. Fourth, there was a sizeable proportion of patients in whom the medical therapies were either changed or discontinued. The high complication and mortality rate in spite of the change in management makes it clear that the lung biopsy procedure needs to be carefully evaluated and, outside of research settings, the risks weighed and explained carefully before proceeding.

Our study is constrained by the limitations of the included studies. Systematic reviews are potentially susceptible to publication bias. We attempted to limit the potential for publication bias by conducting an extensive search for all relevant publications reporting prevalence or providing data from which prevalence could be calculated. We could not conduct a regression analysis based on comorbidities in view of the non-availability of data in the included studies.

## 5. Conclusions

Surgical lung biopsy in patients with ARDS admitted to critical units changes management in a significant number of patients. However, despite the changes in management, mortality rates did not improve. Surgical lung biopsy is associated with significant rates of complications. Considering the overall results, the benefits of surgical lung biopsy in ARDS patients are questionable.

### Take Home Message

- Lung biopsy in ARDS patients leads to a change in management in 3/4 of patients.
- Despite a change in management and pathology-directed treatment, there was significant mortality in ARDS patients who had a lung biopsy.
- High rates of complications from lung biopsy were observed in ARDS patients who had a lung biopsy.
- The decision for lung biopsy in ARDS patients should be individualized considering the complications and mortality rates.

## Figures and Tables

**Table 1:** Demographics and baseline characteristics of the studies. n: number; SD: standard deviation. PEEP: peak end expiratory pressure; PaO_2_/FiO_2_: partial pressure of arterial oxygen/fraction (percent) of inspired oxygen.

Study	Country	Study Type	*n*	Age (Mean ± SD)	Males *n* (%)	PaO_2_/FiO_2_ (mmHg) Mean ± SD	PEEP (cm H_2_O), Mean ± SD
Ortiz 2018 [14]	Columbia	Retrospective	15	42 ± 18	7 (47)	121 ± 0	13 ± 0
Philipponnet 2018 [15]	France	Retrospective	46	69 ± 5	33 (71)	180 ± 89	-
Gerard 2018 [16]	Belgium	Retrospective	51	65 ± 7	28 (55)	128 ± 0	9 ± 2
Almotairi 2016 [17]	Canada	Retrospective	76	63 ± 4	41 (54)	136 ± 24	-
Guerin 2015 [18]	France	Retrospective	83	64 ± 6	35 (56)	-	-
Depuydt 2013 [19]	Belgium	Retrospective	60	62 ± 14	36 (60)	189 ± 0	8 ± 1
Melo 2009 [20]	Portugal	Retrospective	19	58 ± 16	10 (53)	171 ± 0	9 ± 0
Charbonney 2009 [21]	Switzerland	Retrospective	19	50 ± 5	11 (58)	119 ± 34	6 ± 3
Baumann 2008 [22]	Germany	Retrospective	27	48 ± 14	12 (44)	188 ±109	-
Arabi 2007 [23]	Saudi Arabia	Retrospective	14	51 ± 19	7 (50)	150 ± 60	8 ± 4
Lim 2007 [24]	South Korea	Retrospective	36	59 ± 0	25 (69)	159 ± 0	11 ± 4
Papazian 2007 [25]	France	Prospective	100	57 ± 17	67 (67)	129 ± 14	10 ± 3
Barbas 2006 [26]	Brazil	Retrospective	12	65 ± 14	5 (42)	157 ± 0	-
Kao 2006 [12]	Taiwan	Retrospective	41	55 ± 17	28 (68)	116 ± 43	11 ± 3
Soh 2005 [13]	Taiwan	Retrospective	32	51 ± 22	24 (75)	163 ± 90	-
Kapala 2005 [27]	Brazil	Retrospective	13	66 ± 0	-	-	-
Monteiro 2005 [28]	Brazil	Retrospective	24	65 ± 0	12 (50)	-	-
Patel 2004 [29]	USA	Retrospective	57	53 ± 18	36 (65)	145 ± 61	10 ± 4
Flabouris 1999 [30]	Australia	Retrospective	24	49 ±16	8 (33)	161 ± 0	-
Papazian 1998 [8]	France	Prospective	36	59 ± 15	-	122 ± 37	10 ± 0
Hughes 1997 [31]	Canada	Retrospective	27	57 ± 0	14 (52)	-	-
Canver 1994 [32]	USA	Retrospective	92	51 ± 5	13 (48)	-	9 ± 1

**Table 2:** Results of meta regression analysis. Univariate and multivariate regression to evaluate confounding effects of age, gender, and study type. CIM: change in management. *p*-Values > 0.05 would indicate no confounding effects of the variables on the results.

	Univariate: Coefficient of Variation (*p*)			Multivariate: Coefficient of Variation (*p*)		
	Age	Male	Study Type	Age	Male	Study Type
Air-leak	0.98 (0.5)	1.01 (0.2)	0.57 (0.1)	0.97 (0.3)	1.02 (0.76)	0.36 (0.06)
Morality	1.02 (0.1)	1.00 (0.6)	0.89 (0.6)	1.01 (0.1)	1.00 (0.9)	0.85 (0.6)
Complications	0.98 (0.4)	1.00 (0.6)	0.87 (0.7)	0.98 (0.3)	1.01 (0.5)	1.20 (0.7)
CIM	1.01 (0.2)	1.00 (0.9)	1.18 (0.4)	1.01 (0.3)	1.00 (0.7)	1.11 (0.7)

## Data Availability

All included studies were publicly available and published in listed journals.

## Supplementary Materials

The following supporting information can be downloaded at: https://www.mdpi.com/article/10.3390/arm90040036/s1, PICOS and Search strategy; Table S1. Quality assessment of the studies as per modified tool for quality assessment for case series; Figure S1. Pooled proportion of Diffuse alveolar damage on lung biopsy.; Figure S2. Pooled proportion of viral infection on lung biopsy; Figure S3. Pooled proportion of bacterial infection on lung biopsy; Figure S4. Pooled proportion of fungal infections on lung biopsy; Figure S5. Pooled proportion of diffuse alveolar hemorrhage on lung biopsy; Figure S6. Pooled proportion of interstitial lung disease on lung biopsy; Figure S7: Pooled proportion of pneumonitis on lung biopsy.; Figure S8. Pooled proportion of vasculitis on lung biopsy; Figure S9. Pooled proportion of cryptogenic organizing pneumonia on lung biopsy; Figure S10. Pooled proportion of hemothorax secondary lung biopsy procedure; Figure S11. Pooled proportion of pneumothorax secondary lung biopsy procedure; Figure S12. Univariate regression: air leak with study type; results were insignificant; Figure S13. Univariate regression: air leak with gender; results were insignificant; Figure S14. Univariate regression: air leak with age; results were insignificant; Figure S15 Univariate regression: change in management. No effects on results were seen from study type; Figure S16 Univariate regression: change in management. No effects on results were seen from gender; Figure S17. Univariate regression: change in management. No effects on results were seen from age; Figure S18. Univariate regression: complication rate. No effects on results were seen from study type; Figure S19. Univariate regression: complication rate. No effects on results were seen from gender; Figure S20. Univariate regression: complication rate. No effects on results were seen from age; Figure S21. Mortality rate. No effects on results were seen from gender; Figure S22. Univariate regression: mortality rate. No effects on results were seen from age.
