# Supplementary material for: Outcome and Post-Surgical Lung Biopsy Change in Management of ARDS: A Proportional Prevalence Meta-Analysis
Source: Adv Respir Med. 2022 Jul 28;90(4):267–78. doi: 10.3390/arm90040036 (PMC9717336; doi:10.3390/arm90040036)

advances in respiratory medicine

MDPI

PICOS and Search strategy

A: PICOS

Study question:
Outcome of open lung biopsy in ARDS patients admitted to intensive care units.

Population: All patients admitted with ARDS to intensive care units
Intervention: Lung biopsy
Comparison: None
Outcome: Survival at discharge, rates of changes in management, pathologic diagnosis from lung biopsy, overall mortality outcome post lung biopsy.
Study type: Proportion meta-analysis

B: Search strategy for outcome of open lung biopsy in ARDS patients admitted to intensive care units

1). Cochrane total: 78

#.	Searches	Results
1	MeSH descriptor: [Respiratory Distress Syndrome, Adult] explode all trees	1384
2	MeSH descriptor: [Biopsy] explode all trees	5693
3	MeSH descriptor: [Lung] explode all trees	4192
4	#2 OR #3	9747
5	#4 AND #1	78
	**Trials**	**78**
	Cochrane Reviews	0
	Cochrane Protocols	0
	Clinical Answers	0

2). OVID Search: 678

#	Searches	Results
1	Lung biopsy.mp.	18668
2	ARDS.mp.	36804
3	Acute respiratory distress syndrome.mp	43668
4	Outcomes.mp.	2213535
5	#2 OR #3	59381
6	#1 AND #5	1831
7	#6 AND #4	678

3). PubMed: 155
("ARDS" OR "Acute respiratory distress syndrome") AND ("Lung biopsy" OR "lung Histopathology")

Total studies: 911
Duplicates: 226
Total studies qualified for abstract review: 685 (Figure 1)

Table S1. Quality assessment of the studies as per modified tool for quality assessment for case series.

Study	Domains	Selection	Ascertainment	Causality	Reporting	Total
Ortiz	✓	1	2,3	4,7	8	6
Philipponnet	✓	1	2,3	4,7	8	6
Almotairi	✓	1	2,3	4,7	8	6
Gerard	✓	1	2,3	4,7	8	6
Arabi	✓	1	2,3	4,7	8	6
Barbas	✓	1	2,3	4,7	8	6
Baumann	✓	1	2,3	4,7	8	6
Canver	✓	1	2,3	4,7	8	6
Depuydt	✓	1	2,3	4,7	8	6
Flabouris	✓	1	2,3	4,7	8	6
Hughes	✓	1	2,3	4,7	8	6
Kao	✓	1	2,3	4,7	8	6
Lim	✓	1	2,3	4,7	8	6
Melo	✓	1	2,3	4,7	8	6
Papazion	✓	1	2,3	4,7	8	6
Papazion	✓	1	2,3	4,7	8	6
Patel	✓	1	2,3	4,7	8	6
Soh	✓	1	2,3	4,7	8	6
kapala 2005	✓	1	2,3	4,7	8	6
Monteiro 2005	✓	1	2,3	4,7	8	6
Charbonney 2009	✓	1	2,3	4,7	8	6
Guerin 2015	✓	1	2,3	4,7	8	6

Figure S1. Pooled proportion of Diffuse alveolar damage on lung biopsy.

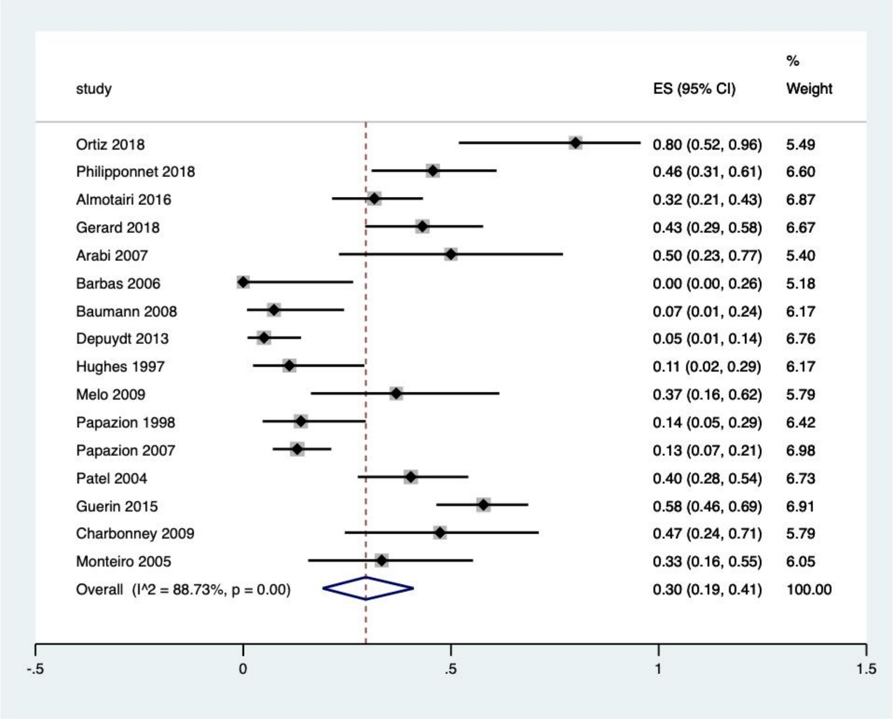

Figure S2. Pooled proportion of viral infection on lung biopsy.

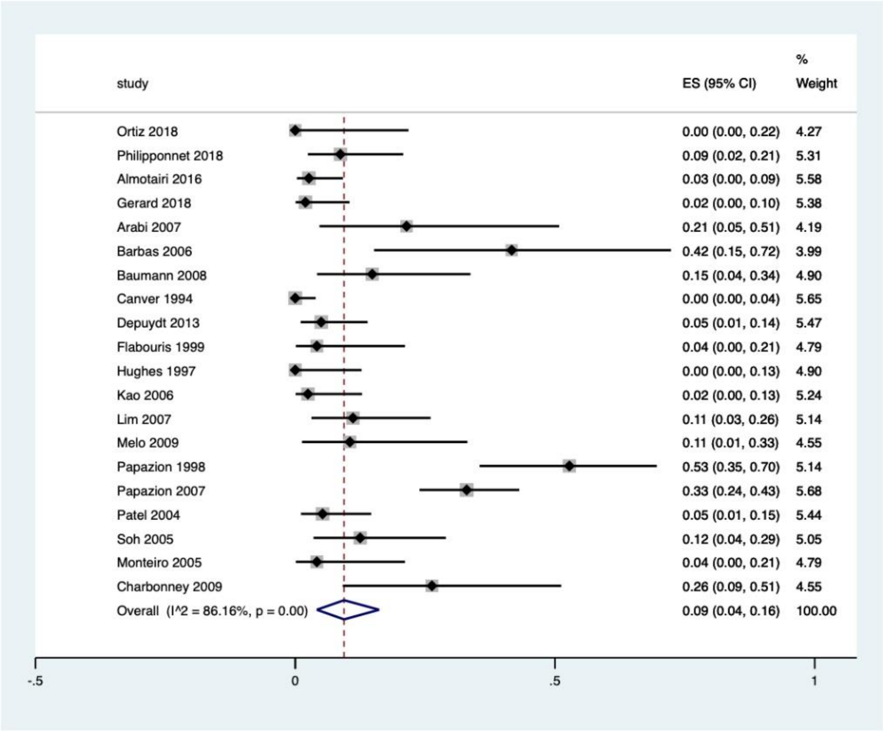

Figure S3. Pooled proportion of bacterial infection on lung biopsy.

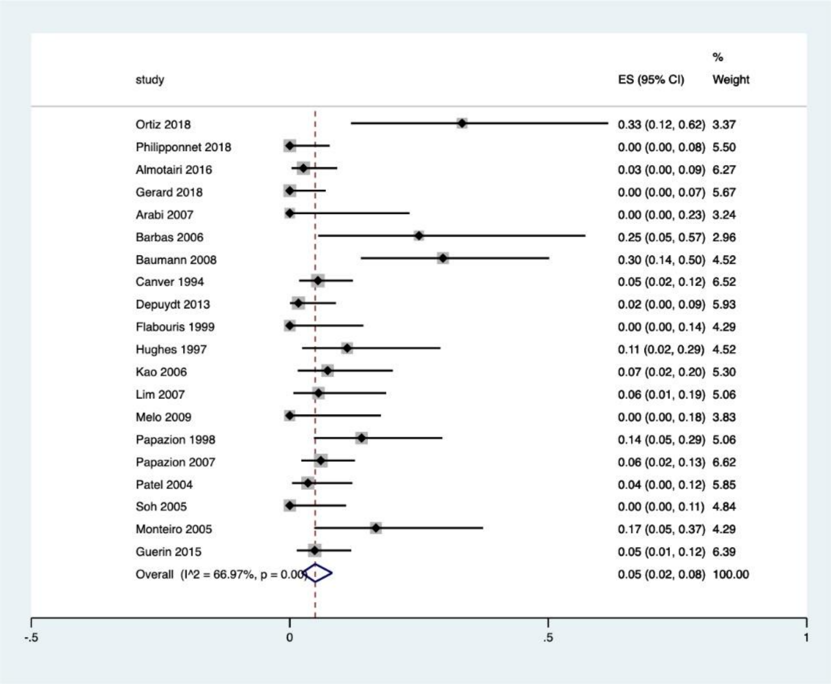

Figure S4. Pooled proportion of fungal infections on lung biopsy.

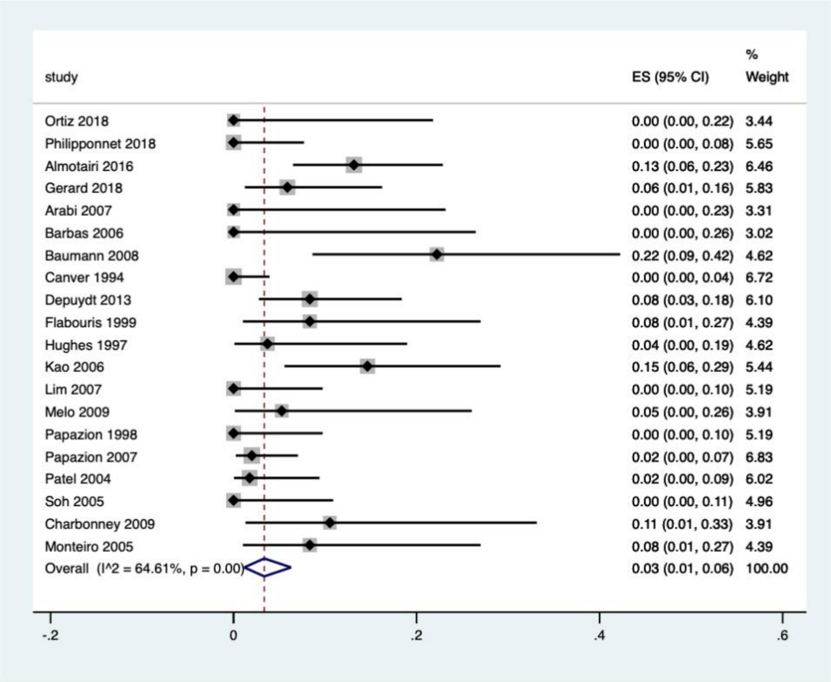

Figure S5. Pooled proportion of diffuse alveolar hemorrhage on lung biopsy.

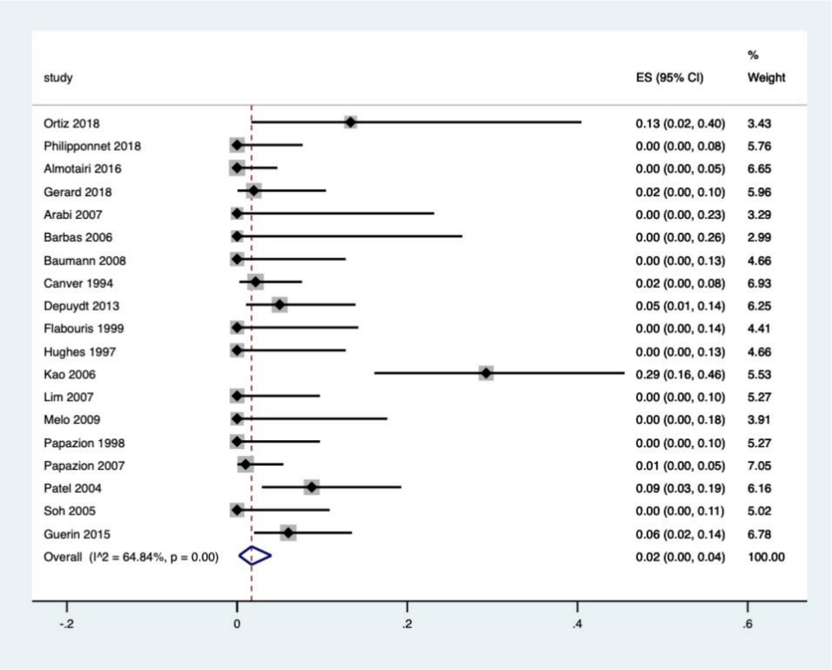

Figure S6. Pooled proportion of interstitial lung disease on lung biopsy.

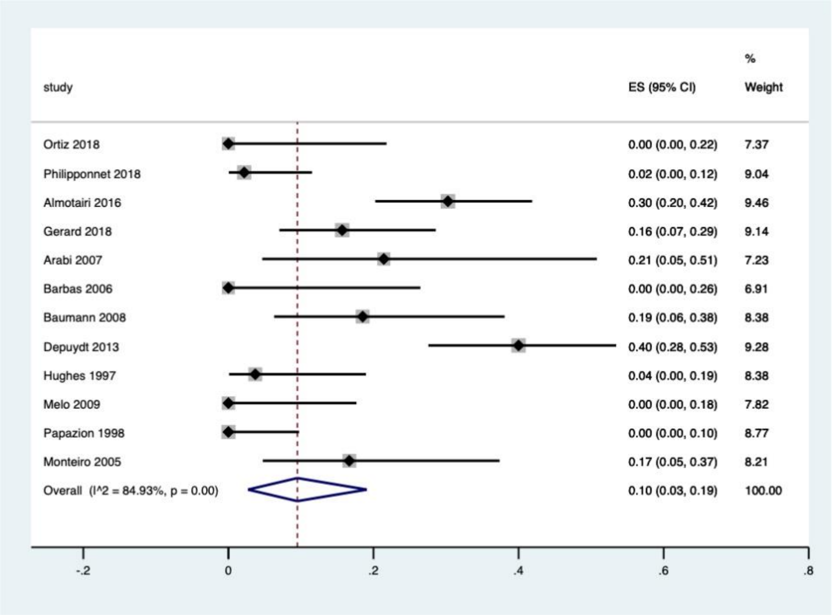

Figure S7: Pooled proportion of pneumonitis on lung biopsy.

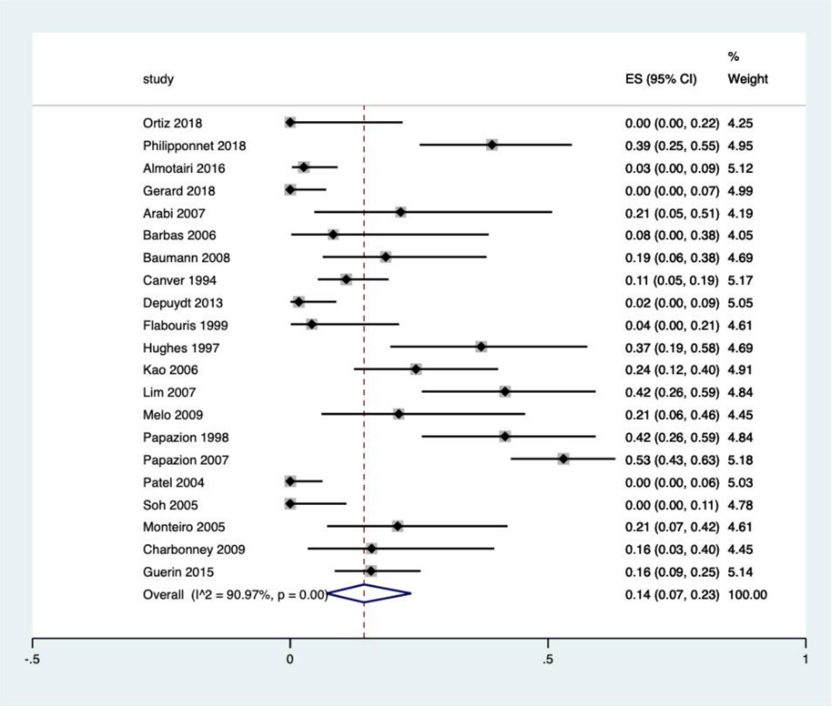

Figure S8. Pooled proportion of vasculitis on lung biopsy.

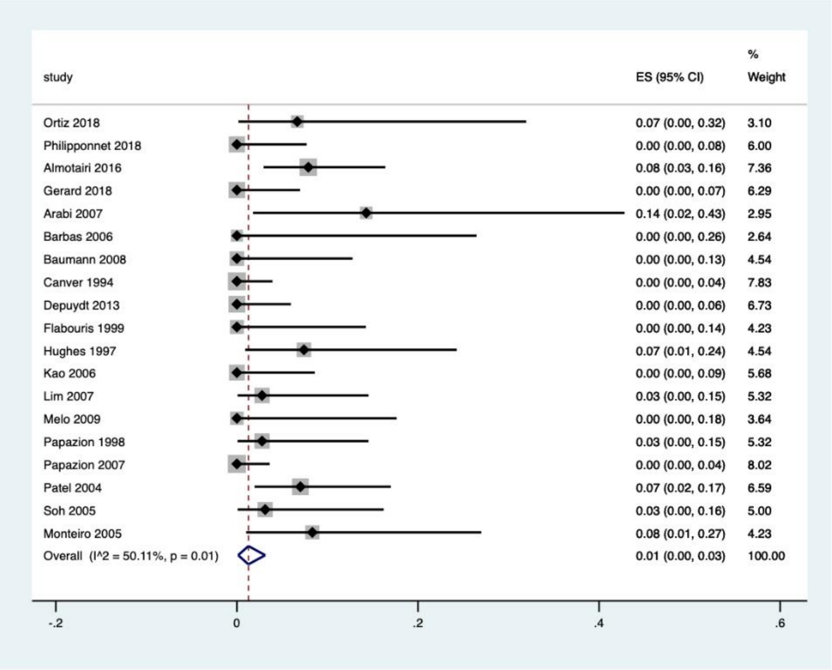

Figure S9. Pooled proportion of cryptogenic organizing pneumonia on lung biopsy.

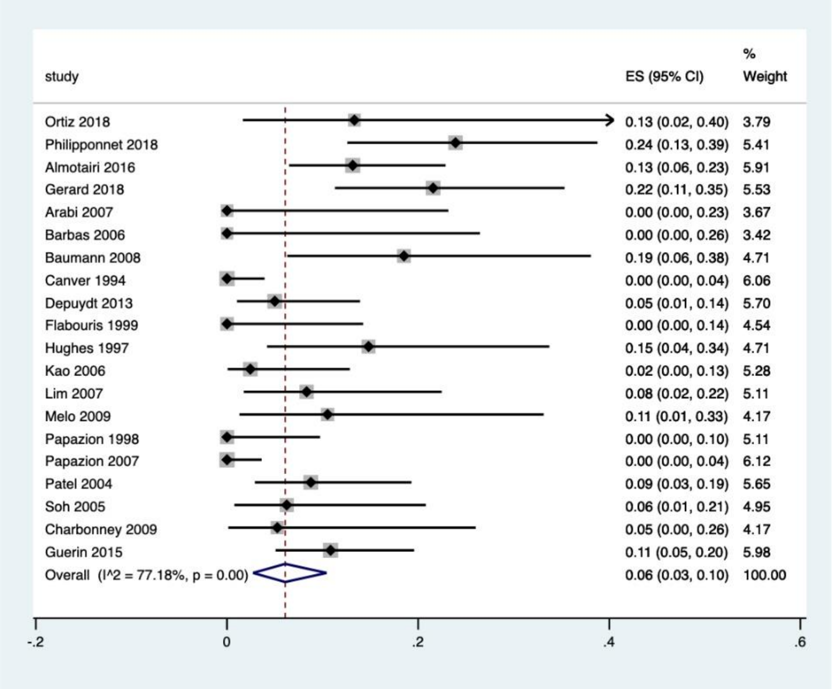

Figure S10. Pooled proportion of hemothorax secondary lung biopsy procedure.

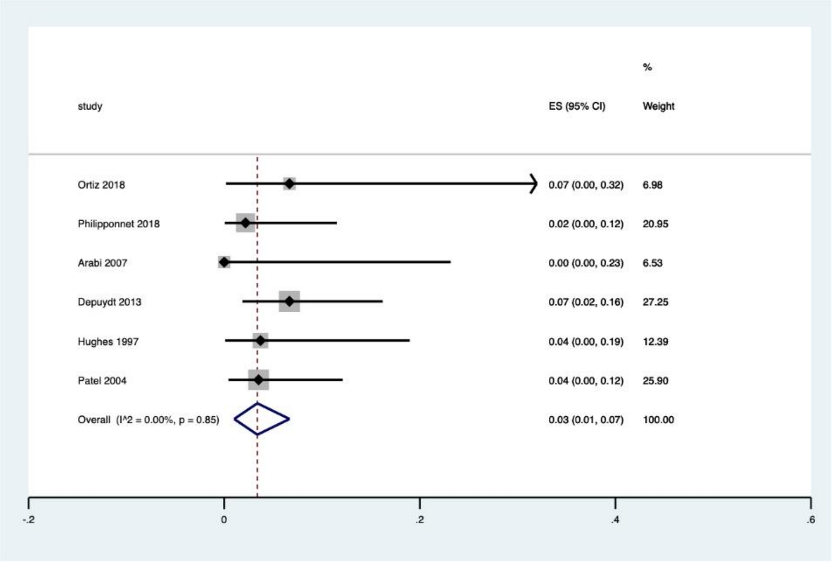

Figure S11. Pooled proportion of pneumothorax secondary lung biopsy procedure.

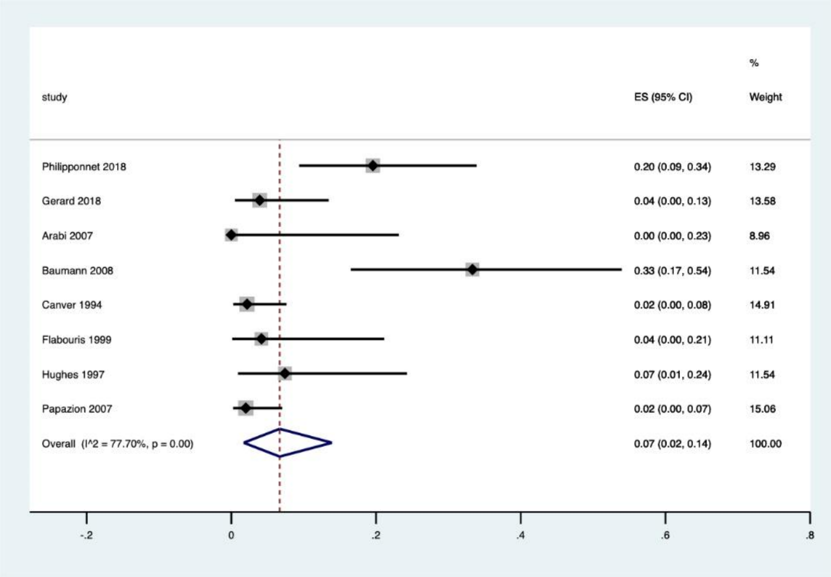

Figure S12. Univariate regression: air leak with study type; results were insignificant.

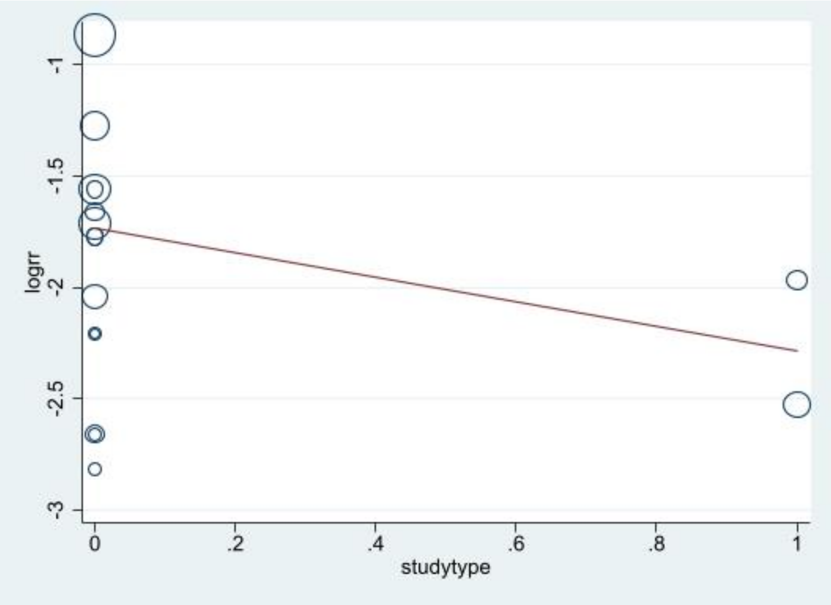

Figure S13. Univariate regression: air leak with gender; results were insignificant.

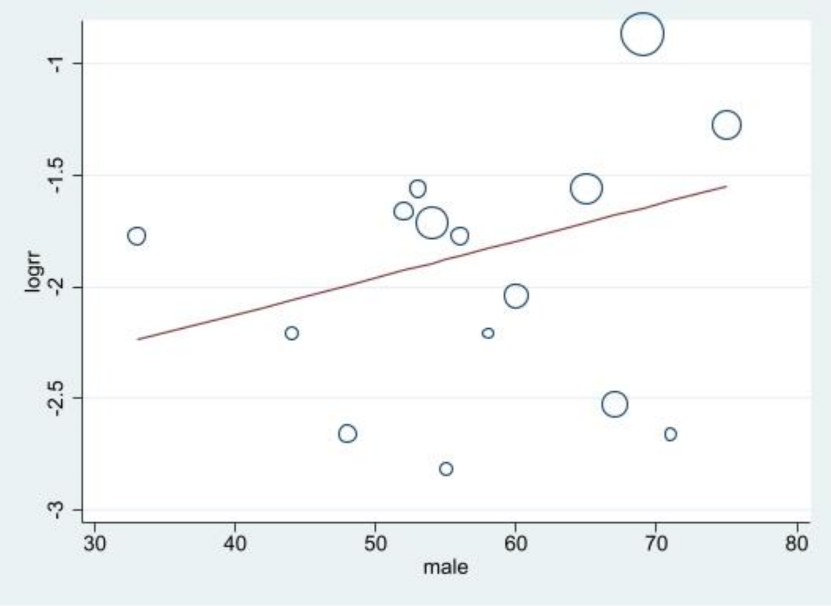

Figure S14. Univariate regression: air leak with age; results were insignificant.

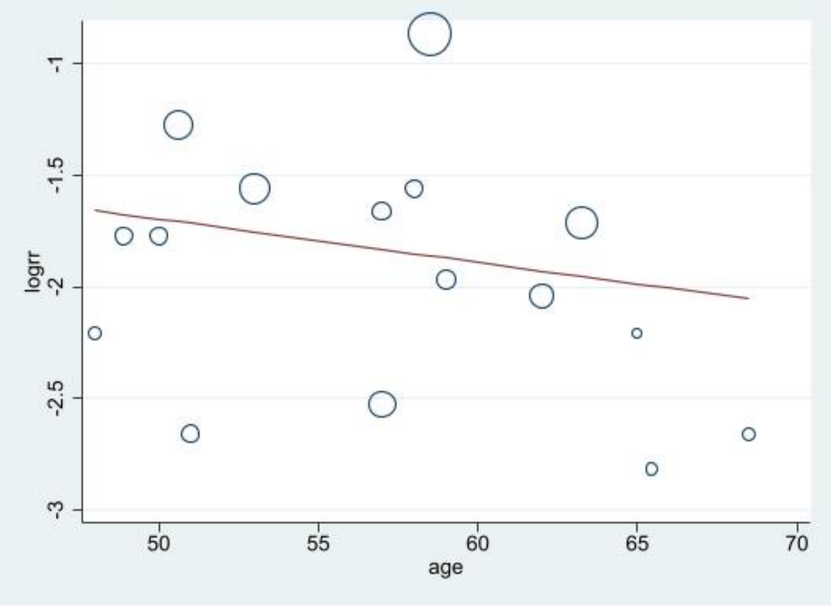

Figure S15 Univariate regression: change in management. No effects on results were seen from study type.

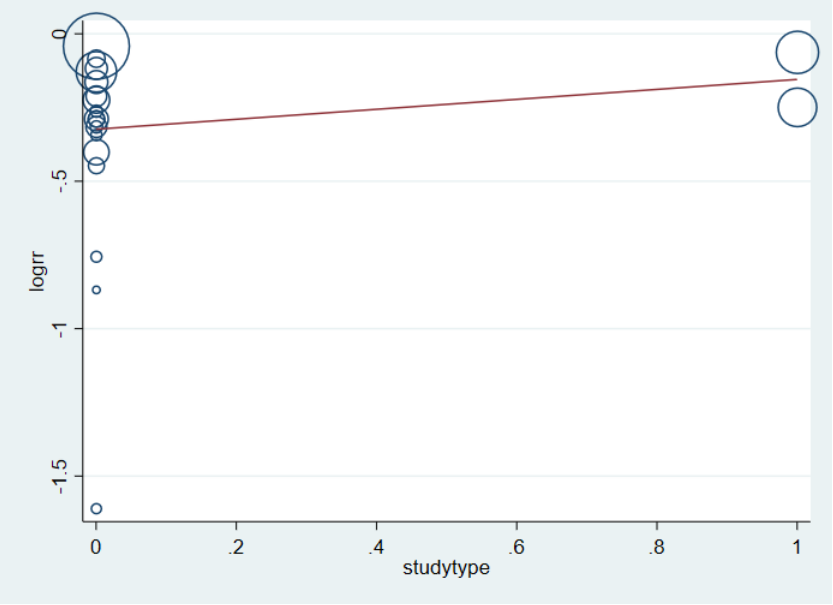

Figure S16 Univariate regression: change in management. No effects on results were seen from gender.

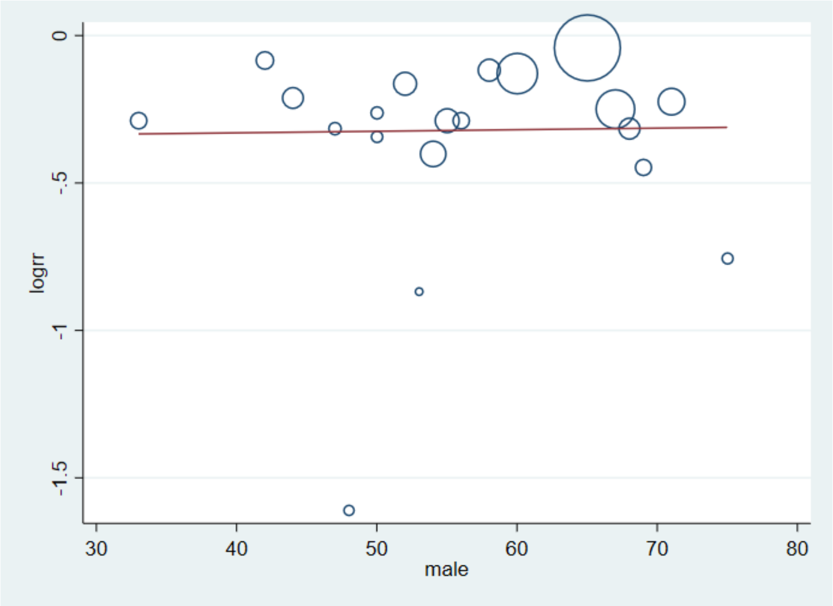

Figure S17. Univariate regression: change in management. No effects on results were seen from age.

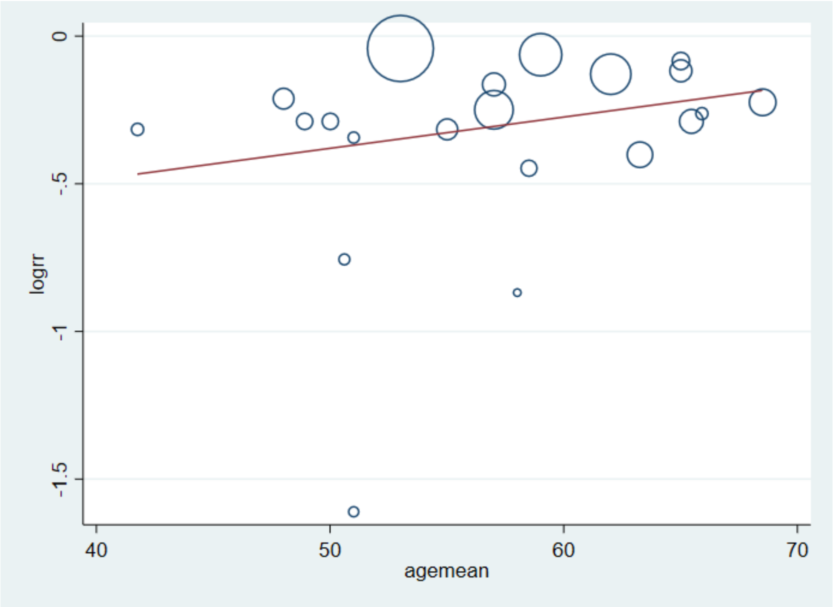

Figure S18. Univariate regression: complication rate. No effects on results were seen from study type.

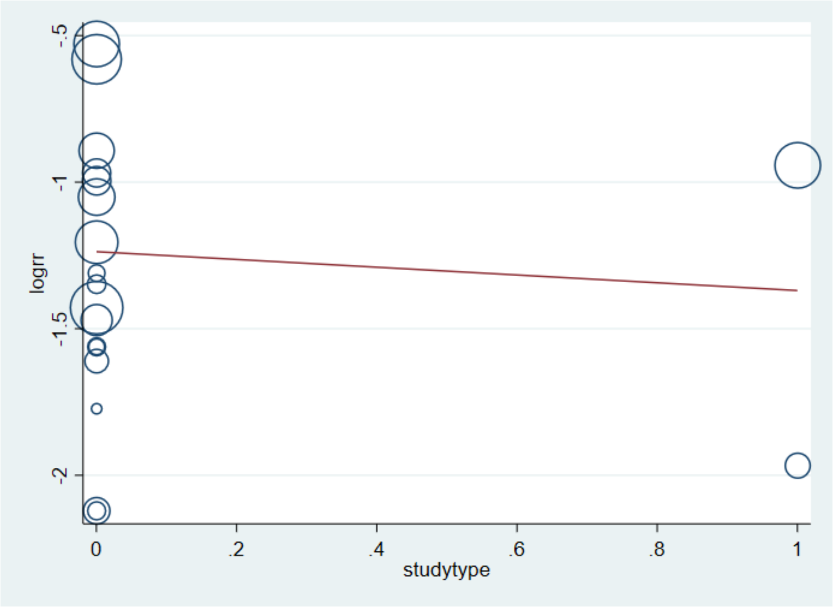

Figure S19. Univariate regression: complication rate. No effects on results were seen from gender.

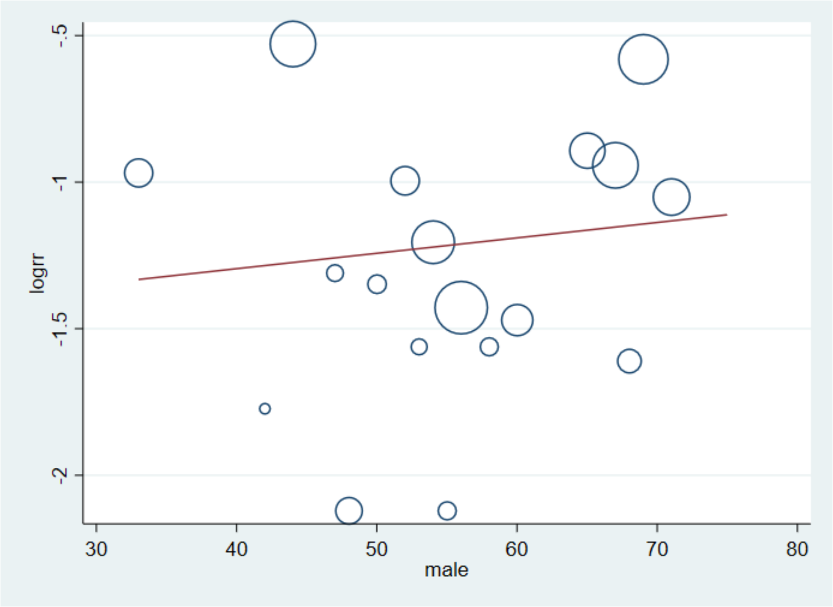

Figure S20. Univariate regression: complication rate. No effects on results were seen from age.

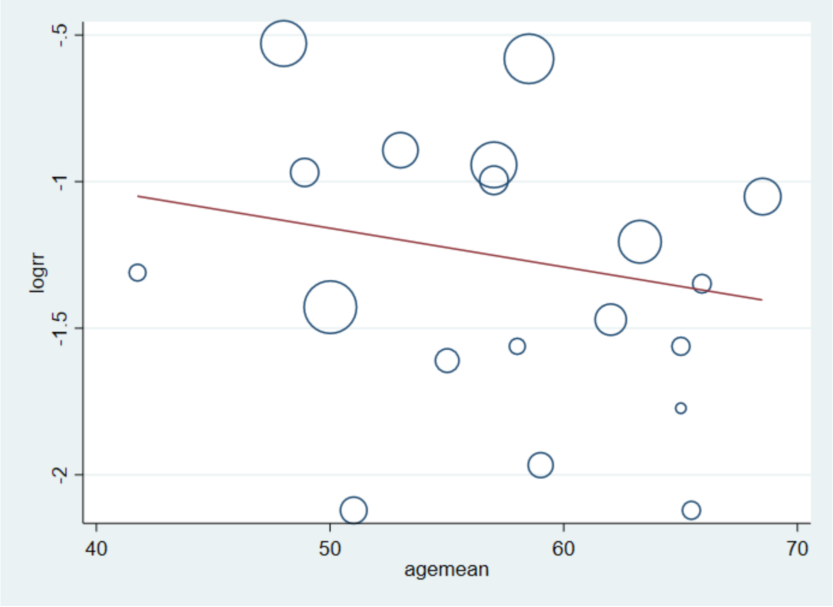

Figure S20. Univariate regression: mortality rate. No effects on results were seen from study type.

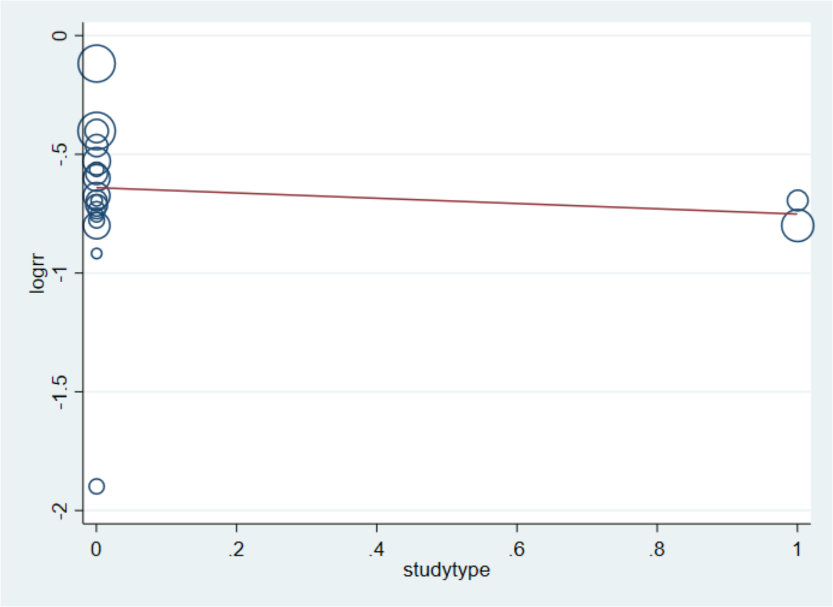

Figure S21. Univariate regression: mortality rate. No effects on results were seen from gender.

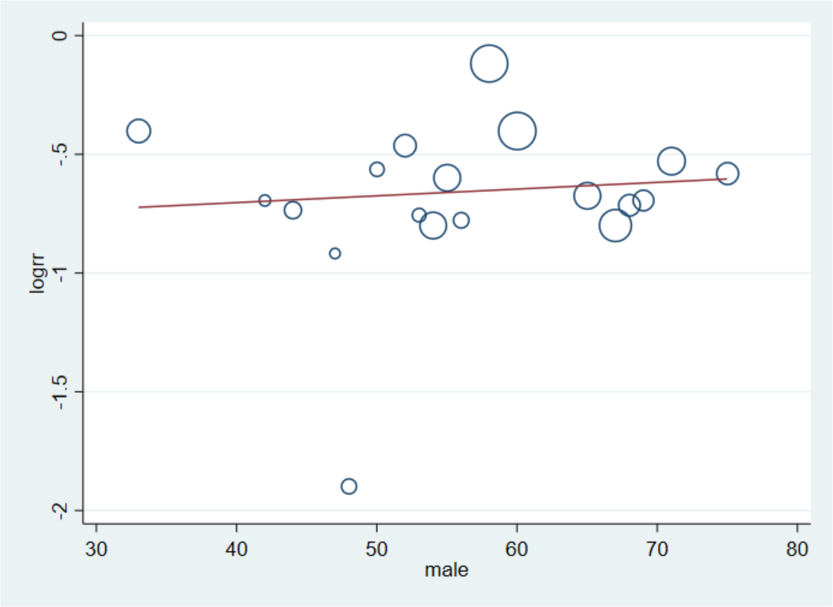

Figure S22. Univariate regression: mortality rate. No effects on results were seen from age.

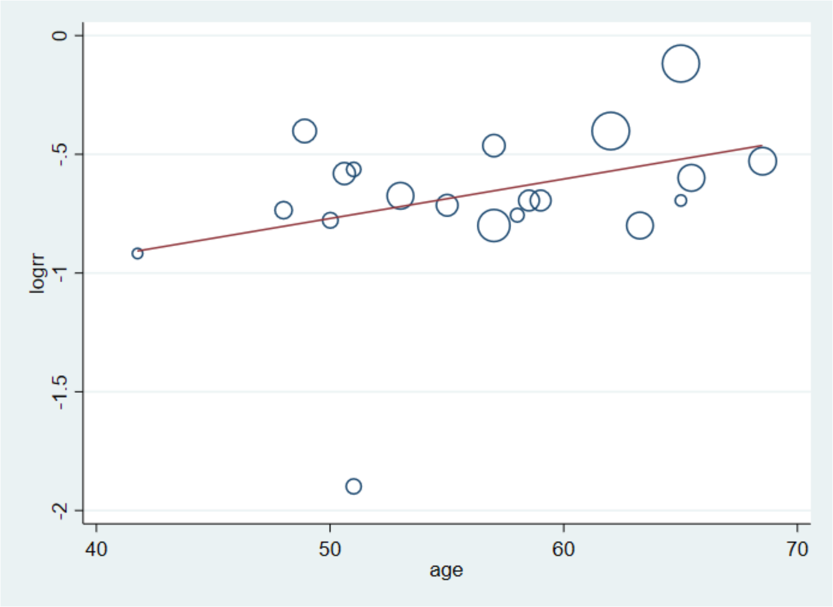

Supplement: Supplementary file 1 [file arm-90-00036-s001.zip › arm-1825092-supplementary.pdf]
